# Effect of Travel on Influenza Epidemiology

**DOI:** 10.3201/eid1906.111864

**Published:** 2013-06

**Authors:** Sanne-Meike Belderok, Guus F. Rimmelzwaan, Anneke van den Hoek, Gerard J.B. Sonder

**Affiliations:** Public Health Service, Amsterdam, the Netherlands (S.-M. Belderok, A. van den Hoek, G.J.B. Sonder);; Academic Medical Centre, Amsterdam (S.-M. Belderok, A. van den Hoek, G.J.B. Sonder);; Erasmus Medical Center, Rotterdam, the Netherlands (G.F. Rimmelzwaan);; National Coordination Centre for Traveler’s Health Advice, Amsterdam (G.J.B. Sonder)

**Keywords:** Influenza, travel, prospective study, attack rate, incidence, viruses, epidemiology

## Abstract

To assess the attack and incidence rates for influenza virus infections, during October 2006–October 2007 we prospectively studied 1,190 adult short-term travelers from the Netherlands to tropical and subtropical countries. Participants donated blood samples before and after travel and kept a travel diary. The samples were serologically tested for the epidemic strains during the study period. The attack rate for all infections was 7% (86 travelers) and for influenza-like illness (ILI), 0.8%. The incidence rate for all infections was 8.9 per 100 person-months and for ILI, 0.9%. Risk factors for infection were birth in a non-Western country, age 55–64 years, and ILI. In 15 travelers with fever or ILI, influenza virus infection was serologically confirmed; 7 of these travelers were considered contagious or incubating the infection while traveling home. Given the large number of travelers to (sub)tropical countries, travel-related infection most likely contributes to importation and further influenza spread worldwide.

International tourism has increased tremendously, with ≈908 million tourist arrivals at airports worldwide in 2007 (*1*). The annual number of travelers from the Netherlands to tropical and subtropical countries, in a population of ≈16 million persons, doubled from ≈1 million in 1999 to ≈2 million in 2007 (*2*). Increased health risks, particularly infectious diseases, are associated with travel. Prospective studies estimate that up to 64% of short-term travelers experience an illness related to travel to (sub)tropical countries (*3*–*5*). In these studies, respiratory tract infections were the second most frequent infectious disease contracted during travel, with attack rates (ARs) up to 26%; fever affected 11%–19.9% of travelers while they were abroad (*3*–*6*). Influenza is one of the most frequently acquired infectious diseases among travelers (*7*). Respiratory tract infections, including influenza in 6% of cases, commonly caused illness among patients admitted to a tertiary-care hospital after they returned from travel (*8*). Among febrile travelers examined at hospitals after return, influenza was diagnosed in up to 15% (*9*–*11*).

The World Health Organization (WHO) estimates that ≈5%–15% of the worldwide population is affected by seasonal influenza viruses annually (*3*). Outbreaks of influenza associated with travel by air, ship, or train indicate that international travelers are at risk for this infection (*12*–*14*) and may introduce novel strains into domestic populations (*15*,*16*). Indeed, in Europe in 2009, >29% of all confirmed cases of influenza A(H1N1)pdm09 virus were related to travel (*17*). Also, of patients admitted to Tan Tock Seng Hospital in Singapore with confirmed influenza A(H1N1) infection, 25% had traveled by plane after onset of illness, and 15% became ill while traveling (*18*).

The incubation period for influenza is 1–5 days, with adults most infectious from 1 day before symptom onset to ≈5–7 days after symptom onset. In healthy adults, a wide range of symptoms occur, varying from classic influenza and mild illness to asymptomatic infection (*19*). Because influenza is highly contagious and has a short incubation period, travel probably contributes considerably to the rapid spread of the virus (*20*).

In temperate climates, influenza is seasonal: most influenza activity occurs in winter, in the Northern Hemisphere during November–March and in the Southern Hemisphere during April–October. In the tropics, however, the destination of many short-term travelers, influenza virus circulates at low levels year-round (*21*,*22*).

Prospective research on influenza during travel is sparse. To our knowledge, the only prospective study that estimated the AR and incidence rate (IR) of influenza among travelers was a cohort study conducted during 1998–2000 (*6*). This study reported that 1.2% of all travelers had a confirmed influenza virus infection, defined as a >4-fold increase in antibody titers, and an influenza incidence of 1.0 per 100 person-months abroad. We prospectively estimated the AR and IR for influenza, risk factors for and proportion of symptomatic and asymptomatic travelers, and geographic areas with particular risk.

## Methods

### Study Population

Persons attending the travel clinic of the Public Health Service Amsterdam during October 2006–October 2007 were recruited for this prospective study. All immunocompetent persons >18 years of age were eligible if they were planning to travel for 1–13 weeks to >1 (sub)tropical countries. We used the definition of the United Nations’ Department of Economics and Social Affairs (*23*) and categorized these countries in 6 regions: South America; Central America and Caribbean; Middle, Western, and Northern Africa; Southern and Eastern Africa; Southeastern and Eastern Asia; and South-central and Western Asia.

All participants were seen by a doctor or nurse who specialized in travel medicine. They received vaccinations, a prescription for antimalarial chemoprophylaxis if required, and oral and written information about how to avoid acquiring travel-related diseases in accordance with national guidelines of the Netherlands for travelers’ health (*24*). No additional information was provided about how to avoid respiratory infections. Influenza vaccination is not routinely advised for healthy travelers (*25*,*26*).

### Survey Methods

Before departure and 2–6 weeks after return, participants donated venous blood samples for serologic testing. A standard questionnaire was used before departure to collect data on sociodemographic characteristics, travel history, and purpose of the travel (tourism, work or education, or visiting friends and/or relatives [VFR]). History of influenza vaccination was not recorded. Participants were given a thermometer and asked to take their temperature if they felt feverish. They were also asked to keep a structured travel diary by recording itinerary, symptoms of disease (such as fever, sore throat, or coughing), and self-treatment or involvement of a doctor. Participants made daily diary entries from the day they arrived at their destination to 1 week after their return, to encompass incubation periods of acute travel-related infections. After travel, the diary was checked for entry gaps and interpretations by a nurse in the participant’s presence. The study protocol was approved by the Medical Ethics Committee of the Academic Medical Center Amsterdam (MEC 06/016).

### Laboratory Methods and Case Definition

All blood samples were immediately stored at 6°C. Blood samples for serologic testing were centrifuged (Hettich Rotixa 50S, APP/407: program 1 [Hettich Rotixa, Beverly, MA, USA] 10 min. 3,000 rpm [210 × *g*]) and frozen at −80°C within 24 h until use. Paired serum samples collected from each study participant before travel and after return were tested simultaneously. The serum samples were tested for antibodies against influenza viruses by using the hemagglutination-inhibition (HI) assay, which was performed in duplicate according to standard methods (*27*,*28*) with turkey erythrocytes and 4 hemagglutinating units of virus propagated in 11-day-old embryonated chicken eggs. For this purpose, vaccine strains IVR-142 (A/Wisconsin/67/05-like [H3N2]), IVR-116 (A/New Caledonia/20/99-like [H1N1]), B/Malaysia/2506/04, and B/Florida/4/06 were used to represent the epidemic strains that circulated worldwide during the study period (*29*). Ferret antiserum raised against the respective vaccine strains were used as positive controls (mean titers for subtype H3N2: 2,560; subtype H1N1: 1,280; strain B/Malaysia: 480; and strain B/Florida: 1,280). For statistical analysis, a titer of 5 was arbitrarily assigned to serum with a titer <10. Titers were transformed to a logarithmic scale, and geometric means were used for further calculations. Because blood samples were collected 2–6 weeks after return of travel, acute influenza virus infections were not expected, and therefore virus isolation and PCR were not used to detect virus in respiratory tract specimens. Pretravel titers of >40 for >1 influenza viruses were defined as protective antibody titers. If the posttravel titer for >1 influenza viruses was >40 and showed a >4-fold increase above pretravel titer, we defined it as a (serologically) confirmed influenza virus infection. Antibodies to influenza B viruses can cross-react and overlap to a certain extent with the other influenza B virus.

Fever was registered when participants reported a body temperature of >38.0°C or when they recorded fever without temperature. In addition to fever, other clinical symptoms were scored as influenza-like illness (ILI) when participants recorded fever with a temperature of >38.0°C or recorded fever without temperature and a sore throat and/or cough according to the WHO definition (*30*). Participants who, in addition to fever, had self-reported “flu” or self-reported “cold” also were included in the ILI definition.

### Data Analysis

Data analysis was performed with SPSS version 19.0.0.1 (2010; IBM, Somers, NY, USA) and Stata version 11 (StataCorp LP, College Station, TX, USA). ARs were calculated by dividing the number of study participants displaying seroconversion for >1 influenza viruses (indicating infection) by the total number of participants at risk. IRs per 100 person-months were calculated by dividing the number of travelers with confirmed influenza virus infections by the total number of travel months in which participants were at risk for infection. For travelers with confirmed influenza virus infection, we used half of their travel duration; for travelers without confirmed influenza virus infection we used their total travel duration. Pearson χ^2^ tests of association were used to compare categorical variables between any 2 groups. Relative risks of confirmed influenza virus infection were expressed as ratios of the IR (IRR). IRRs and 95% CIs were obtained by fitting Poisson regression models that predicted the incidence of confirmed influenza virus infection according to the variables sex, age, country of birth, previous travel, purpose of travel, travel destination, and fever or ILI as a variable for symptoms. The multivariable model included all variables in a backward stepwise regression. Participants with any missing values were omitted from the regression models. We chose the backward stepwise approach to ensure inclusion of variables involved in suppressor effects and thus to reduce the risk of making a type II error. A p value <0.05 was considered statistically significant.

## Results

### Study Population

Originally, we recruited 1,273 immunocompetent persons who intended to travel to (sub)tropical countries. Of these, 83 (7%) were excluded: 23 had their travel arrangements cancelled; 42 were lost to follow-up; and 18 did not have enough enough serum collected to perform influenza serology. Of the remaining 1,190 persons, 510 (43%) were male (Table 1, Appendix, wwwnc.cdc.gov/EID/article/19/6/11-1864-T1.htm). Median age was 37 years (interquartile range [IQR] 28–51 years). Most (966 [81%]) previously had visited (sub)tropical countries. Most (1,103 [93%]) participants were born in a Western country; 1,017 (86%) traveled for holiday; 100 (8%) traveled for work or education, and 73 (6%) were VFR. Median travel duration was 21 days (IQR 15–28 days). The most frequently visited continent was Asia (46%); 27% traveled to Latin America and 24% to Africa.

**Table 1:** Infection with >1 influenza type in 1,190 travelers from the Netherlands to (sub)tropical countries, October 2006–October 2007*

Characteristic	Travelers at risk, no. (%)	Travelers with infection†		Person-months of travel	IR, %‡ (95% CI)	Univariable analysis			Multivariable analysis	
		No.	AR, % (95% CI)			IRR (95% CI)	p value		IRR (95% CI)	p value
All	1,190 (100)	86	7.2 (5.9–8.8)	969	8.9 (7.1–10.9)					
Sex										
M	510 (43)	31	6.1 (4.2–8.4)	404	7.7 (5.3–10.8)	1.0	0.286			
F	680 (57)	55	8.1 (6.2–10.4)	565	9.7 (7.4–12.6)	1.3 (0.8–2.0)				
Age group, y										
<25	140 (12)	8	5.7 (2.7–10.6)	151	5.3 (2.5–10.1)	1.00	0.098		1.0	
25–34	381 (32)	22	5.8 (3.8–8.5)	315	7.0 (4.5–10.4)	1.3 (0.6–3.0)			1.3 (0.6–2.9)	0.558
35–44	223 (19)	21	9.4 (6.1–13.8)	168	12.5 (7.9–18.8)	2.4 (1.0–5.3)			2.2 (1.0–4.9)	0.063
45–54	226 (19)	13	5.8 (3.2–9.4)	165	7.9 (4.4–13.1)	1.5 (0.6–3.6)			1.3 (0.5–3.2)	0.538
55–64	165 (14)	17	10.3 (6.3–15.7)	124	13.7 (8.3–21.5)	2.6 (1.1–6.0)			2.6 (1.1–6.1)	**0.025**
>65	55 (5)	5	9.1 (3.4–19.0)	46	10.9 (4.0–24.1)	2.1 (0.7–6.3)			1.9 (0.6–5.8)	0.259
Region of birth										
Western	1,103 (93)	72	6.5 (5.2–8.1)	905	8.0 (6.3–10.0)	1.0	**0.006**		1.0	
Nonwestern										
Africa	12 (1)	3	25.0 (6.8–54.1)	11	27.3 (6.9–74.2)	3.4 (1.1–10.8)			3.7 (1.2–11.9)	**0.029**
Latin America	47 (4)	9	19.1 (9.8–32.2)	31	29.0 (14.2–53.3)	3.7 (1.8–7.4)			3.8 (1.9–7.8)	**<0.001**
Asia	28 (2)	2	7.1 (1.2–21.7)	22	9.1 (1.5–30.0)	1.1 (0.3–4.6)			1.1 (0.3–4.4)	0.926
Previous travel to(sub)tropical country										
Never	224 (19)	11	4.9 (2.6–8.4)	180	6.1 (3.2–10.6)	1.0	0.140			
1–5 times	687 (58)	49	7.1 (5.4–9.2)	573	8.6 (6.4–11.2)	1.4 (0.7–2.7)				
>6 times	279 (23)	26	9.3 (6.3–13.2)	217	12.0 (8.0–17.3)	2.0 (1.0–4.0)				
Primary purpose of travel										
Tourism	1,017 (85)	73	7.2 (5.7–8.9)	826	8.8 (7.0–11.1)	1.0	1.000			
Work or education	100 (8)	8	8 (3.8–14.6)	86	9.3 (4.3–17.7)	1.1 (0.5–2.2)				
Visiting friends and/or relatives	73 (6)	5	6.9 (2.6–14.5)	57	8.8 (3.2–19.4)	1.0 (0.4–2.4)				
Travel destination										
Latin America										
South America	202 (17)	13	6.4 (3.6–10.5)	167	7.8 (4.3–13.0)	1.0	0.373			
Central America and Caribbean	120 (10)	9	7.5 (3.7–13.3)	78	11.5 (5.6–21.2)	1.5 (0.7–3.5)				
Africa										
Southern and Eastern Africa	181 (15)	12	6.6 (3.6–11)	147	8.2 (4.4–13.9)	1.1 (0.5–2.3)				
Middle, Western, Northern Africa	110 (9)	10	9.1 (4.7–15.6)	75	13.3 (6.8–23.8)	1.7 (0.8–3.9)				
Asia										
Southeastern and Eastern Asia	416 (35)	25	6.0 (4.0–8.6)	365	6.9 (4.5–10.0)	0.9 (0.5–1.7)				
South-central and Western Asia	125 (11)	14	11.2 (6.5–17.7)	97	14.4 (8.2–23.6)	1.9 (0.9–4.0)				
More regions	33 (3)	3	9.1 (2.4–22.8)	37	8.1 (2.1–22.1)	1.1 (0.3–3.7)				
More continents	3 (<1)	0	0							
Symptoms										
Fever										
No	1,073 (90)	71	6.6 (5.2–8.2)	854	8.3 (6.5–10.4)	1.0	0.131			
Yes	117 (10)	15	12.8 (7.6–19.8)	115	13.0 (7.6–21.0)	1.6 (0.9–2.7)				
ILI§										
No	1,150 (97)	77	6.7 (5.4–8.3)	930	8.3 (6.6–10.3)	1.0	**0.010**		1.0	**0.004**
Yes	40 (3)	9	22.5 (11.6–37.3)	38	23.7 (11.6–43.5)	2.8 (1.4–5.7)			2.8 (1.4–5.5)	

*AR, attack rate; IR, incidence rate; IRR, incidence rate ratio; ILI, influenza-like illness. **Boldface** indicates significance (p<0.05). †Travelers with confirmed influenza infection for >1 influenza virus. ‡Per 100 person-months. §Travelers with ILI are a subgroup of travelers with fever.

Median time between first blood sample and travel departure was 24 days ([IQR 12–37 days); median time between return and second blood sample was 23 days (IQR 19–27 days). Of all participants, 592 (50%) donated the first blood sample, and 493 (41%) donated the second blood sample during the influenza season in the Netherlands.

### Protective Antibody Titers

Of the 1,190 travelers, 839 (71%) had protective antibody titers; 633 (75%) were positive for A(H3N2), 328 (39%) for A(H1N1), 307 (37%) for B/Malaysia, and 370 (44%) for B/Florida (Table 2). Of the 839 travelers with protective antibody titers, 243 (29%) were immune to 2 influenza viruses, 140 (17%) for 3 influenza viruses, and 92 (11%) for 4 influenza viruses.

**Table 2:** Increase in titer and symptoms of influenza-like illness in 1,190 travelers from the Netherlands to (sub)tropical countries, October 2006–October 2007

Strain, subtype, pretravel titer	No. travelers, n = 1,190	Titer >4-fold, no. (%), n = 120*	Influenza-like illness, no. (%), n = 11
A			
H3N2			
<40	557	32 (6)	6 (19)
>40†	633	12 (2)	1 (8)‡
H1N1			
<40	862	24 (3)	1 (4)
>40†	328	5 (2)	0
B/Malaysia			
<40	883	20 (2)	2 (10)
>40†	307	3 (1)	0
B/Florida			
<40	820	22 (3)	1 (5)
>40†	370	2 (1)	0

*>4-fold rise between pretravel and posttravel titer and posttravel titer >40. †>40: protective antibodies. ‡Symptoms probably caused by asthma.

### Confirmed Influenza Virus Infections

Eighty-six travelers had a confirmed influenza infection caused by >1 viruses (Table 1). The AR was 7% (95% CI 6%–9%). Of these 86 travelers, 72 (84%) were born in a Western country; 31 (36%) were male, and 75 (87%) had traveled previously. Median age was 43 years (IQR 29–55 years). The travel destination with the highest AR and IR was South-central and Western Asia. The IR for serologically confirmed influenza virus infection per 100 person-months was 8.9 (95% CI 7.1–10.9).

Of the 86 participants, 66 (77%) displayed a rise in antibody titer against 1 influenza virus; 11 (13%) against 2 viruses; 4 (5%) against 3 viruses, and 5 (6%) against all 4 viruses, making a total of 120 recent infections. Of all 120 confirmed influenza virus infections, 44 (37%) were caused by A(H3N2); 29 (24%) by A(H1N1); 23 (19%) by B/Malaysia, and 24 (20%) by B/Florida (Table 2). Twenty-two (18%) infections occurred in travelers who had protective antibodies against the same strain before travel.

### Symptoms

Of the 1,190 travelers, 117 (10%) had fever with a median temperature of 38.6°C (range 38.0°C–41.3°C); illness of 40 (3%) met the definition for ILI. Influenza virus infection was confirmed in 15 (13%) of the 117 travelers with fever and in 9 (23%) of the 40 travelers with ILI; 6 travelers had only fever, but their illness did not meet the ILI definition.

The AR of symptomatic (ILI) confirmed influenza virus infection for all travelers was 0.8% (95% CI 0.4%–1.4%) with an IR of 0.9 per 100 person-months (95% CI 1.2–3.2). In the analysis of travelers with symptomatic confirmed influenza virus infection, no determinants were found. For none of the 4 viruses did we find an association between level of posttravel titer and ILI. Only 1 traveler had protective antibody titers against all 4 influenza viruses, displayed a confirmed A(H3N2) infection, and also displayed ILI. This 28-year-old woman was known to have asthma and to have been hospitalized and treated for symptoms of asthma with acetylcysteïne, salbutamol, and amoxicillin.

Of the 9 travelers with symptomatic confirmed influenza virus infection, 7 (78%) sought medical attention abroad, but aside from the asthmatic woman, no hospitalizations were recorded. Of the 15 travelers with confirmed influenza virus infection with fever or ILI, in 7 (47%) symptoms started within 1 week before returning home or shortly after return.

### Independent Risk Factors for Influenza Virus Infection

Age, country of birth, and ILI were independently associated with confirmed influenza virus infection (Table 1). IRR was significantly higher for persons 55–64 years of age (IRR 2.6 (95% CI 1.1–6.1) than for persons <25 years; IRRs were significantly higher for travelers born in an African (IRR 3.7 [95% CI 1.4–5.5]) or Latin American (IRR 3.8 [95% CI 1.9–7.8]) country than for persons born in a Western country (IRR 3.8 [95% CI 1.9–7.8]); and IRR was significantly higher for travelers with ILI (IRR 2.8 [95% CI 1.4–5.5]) than for travelers without ILI. Travel duration was not associated with influenza virus infection (Pearson χ^2^, p = 0.808). For none of the 4 viruses did we find significant associations between confirmed influenza virus infection and travel destination.

## Discussion

This prospective study with short-term travelers to (sub)tropical countries supports earlier studies showing that influenza is 1 of the most frequently acquired infectious travel-related diseases (*7*). The AR for confirmed influenza virus infections was 8%; the IR 8.9 per 100 person-months. The AR and IR of symptomatic (ILI) confirmed influenza virus infection for all travelers was 0.8% and 0.9 per 100 person-months, respectively.

The prospective study by Mutsch et al. (*6*) found an AR for infection with a >4-fold increase in antibody titers of 1.2% and an incidence of 1.0 per 100 person-months of travel, lower than the overall AR and IR in our study. Mutsch et al. found an AR for symptomatic infection (defined as fever alone) of 0.9%; in our study, AR for confirmed influenza virus infection with fever was 2% (95% CI 0.7%–2%), which is broadly similar to results of Mutsch et al.

However, comparing these 2 studies is difficult because of different study methods and circumstances. First, the characteristics of the populations differed. Mutsch et al. included travelers >12 years of age who had travel duration <6 months for almost 3 total influenza seasons, and this could have affected the AR and IR. Further, we serologically tested the entire study population, whereas they tested a subgroup of travelers with febrile illness and a matched group of travelers without febrile illness, possibly leading to an underestimation of influenza infections. They also defined a group of probable cases by a 2.0- to 3.9-fold increase in antibody titer. In our study, a >4-fold titer rise in antibody was used as serologic evidence for influenza infection, which is an accepted threshold in the field (*31*). Both studies showed that participants born in African and Latin American countries are more likely to have contracted influenza virus infections during travel (p = 0.029 and p<0.001, respectively) than those born in Western countries. Also, in other studies an association was found between country of birth and risk for certain other infections (*8*,*32*,*33*). Possibly VFR travelers have a higher risk for influenza because they tend to have closer contact with the local population (*8*,*34*,*35*). The GeoSentinel surveillance network (*8*) showed that VFRs and a trip duration of >30 days were associated with influenza. In our study and the study by Mutsch et al. (*6*), travel duration was not significantly associated with confirmed influenza virus infection (p = 0.808). Mutsch et al. found the Indian subcontinent to be a higher risk area (*6*). We also found South-central and Western Asia to have the highest AR and IR of all regions (11%; 14.4/100 person-months), although these rates did not differ significantly from other destinations. In our study, the IRR was significantly higher for persons 55–64 years of age than for persons <25 years, for which we do not have an explanation.

In 3 of the travelers who had confirmed influenza virus infection with fever or ILI, symptoms started within 1 week before they returned home; they were thus considered to be contagious during the flight. In 4 travelers who had confirmed influenza virus infection with fever or ILI, the symptoms started within 1 week after return. These 7 travelers probably imported an influenza virus that could spread in the Netherlands. In the tropics, influenza viruses circulate throughout the year (*21*,*22*). That travel occurs year-round suggests that influenza viruses are imported continuously and spread to other regions of the world. Because 7 of 1,190 travelers in our study could have imported influenza virus into the Netherlands, the ≈2 million travelers from the Netherlands who visit (sub)tropical countries could theoretically represent ≈12,000 persons importing influenza viruses annually. Because asymptomatic travelers also could be infectious, the number of travelers who import influenza virus is probably underestimated. Indeed, evidence suggests that influenza A(H3N2) viruses originate in Southeastern and Eastern Asia and are spread continuously, causing epidemics worldwide (*16*).

Only 1 traveler with protective antibody titers to all 4 influenza viruses had a confirmed subtype H3N2 infection and ILI. Although we did not register influenza vaccination status, protective antibodies in this traveler probably resulted from vaccination. This traveler had asthma, and all asthma patients are offered free influenza vaccinations by their general practitioner annually. The influenza vaccine contained the same 4 strains for which we tested. The traveler was hospitalized during travel with asthma symptoms. Symptoms could have been part of the asthma spectrum and might not have been caused by the influenza virus infection. In that case, none of the participants in our study with protective antibody titers had a symptomatic influenza virus infection (i.e., all symptomatic infections occurred in susceptible travelers) (Table 2). Many travelers had pretravel protective antibody titers to >1 influenza viruses, which may be explained by a history of infection with influenza viruses and not by influenza vaccination because, in the Netherlands, influenza vaccination is not routinely advised for healthy travelers.

Our study has several strengths. The prospective nature of the study enabled an estimation of the AR and IR of confirmed influenza virus infection with ILI or fever and of asymptomatic confirmed influenza virus infections. The daily diary entries, which minimize recall bias, provide a good record of symptoms during travel. A high percentage (90%) of study participants with fever used the thermometer that was offered before travel to measure their body temperature. The HI assay is the method of choice for seroepidemiologic surveys because it is relatively easy to perform and can be used to detect recent infections using preconvalescent-phase and convalescent-phase serum samples (*6*,*36*–*38*).

Our study also has some limitations. First, because blood samples were taken some time before and after travel, participants could have been infected with influenza virus in the Netherlands. About half of the follow-up time was spent during the influenza season in the Netherlands and not abroad, which might have resulted in an overestimation of travel-related influenza. Mutsch et al. had the same limitation. In Europe, the 2006–07 influenza season began in November and peaked in January and was reported to be generally mild. In the Netherlands, the 2006–07 influenza epidemic, with a maximum clinical influenza activity of 8.2/10,000 inhabitants/week, was among the 3 smallest registered since 1969 (*39*), suggesting that the number of influenza virus infections contracted in the Netherlands might have been low. Because the Southern Hemisphere also had mild influenza activity during our study period (*40*), the higher incidence of confirmed influenza virus infections in our study compared with that of Mutsch et al. is difficult to explain, possibly because of differences in the sensitivity of the HI assays used. Furthermore, the contribution of other infectious diseases to disease symptoms cannot be excluded. Although influenza B/Malaysia/2506/04 and B/Florida/4/06 viruses belong to different lineages, antibodies against these viruses may cross-react to a certain extent. Therefore, seropositivity to these viruses should not be considered independent events. Because we found 120 infections in 86 travelers, including 23 B/Malaysia and 24 B/Florida virus infections, possible cross-reactions could have led to overestimation of the number of influenza B virus infections.

Vaccination of all travelers against influenza has been discussed (*22*). In Canada the Committee to Advise on Tropical Medicine and Travel recommends influenza vaccination to all healthy travelers. WHO recommends annual influenza vaccination only for travelers who have conditions that place them at high risk for complications of influenza. In the Netherlands, as in many other countries, influenza vaccination is already recommended for these risk groups, irrespective of travel. Because travel is not a risk factor for severe disease, we believe that there is no need to advise influenza vaccinations to all healthy travelers. In case of an influenza pandemic with a new strain, vaccination could play a role in the control of outbreak, but not in the beginning, because a new vaccine will not readily be available (*22*).

In conclusion, short-term travelers to (sub)tropical regions contract influenza regularly, which is probably a major factor in the epidemiology of influenza. Fifty percent of travelers with symptomatic influenza could have imported the virus into the Netherlands. Because travelers often visit (sub)tropical regions, where influenza viruses continuously circulate, after contracting the disease they become vectors that further spread the virus worldwide.
